# A Subset of Osteosarcoma Bears Markers of CXCL12‐Abundant Reticular Cells

**DOI:** 10.1002/jbm4.10596

**Published:** 2022-01-11

**Authors:** Branden R Sosa, Ziqi Wang, John H Healey, Meera Hameed, Matthew B Greenblatt

**Affiliations:** ^1^ Department of Pathology and Laboratory Medicine Weill Cornell Medicine New York NY USA; ^2^ Orthopaedic Service, Department of Surgery Memorial Sloan Kettering Cancer Center New York NY USA; ^3^ Department of Pathology Memorial Sloan Kettering Cancer Center New York NY USA

**Keywords:** PRIMARY TUMORS OF BONE AND CARTILAGE, CANCER, STROMAL/STEM CELLS, CELLS OF BONE, OSTEOBLASTS, CELL/TISSUE SIGNALING—TRANSCRIPTION FACTORS

## Abstract

Currently, the cell of origin for osteosarcoma or other primary skeletal tumors is largely unknown. Recent reports identifying specific cell types comprising bone now newly enable investigation of this topic. Specifically, CXC motif chemokine 12 (CXCL12)‐abundant reticular (CAR) cells are a specific skeletal stromal cell type that orchestrate the bone marrow microenvironment through cross‐talk with hematopoietic and endothelial cells and a likely candidate cell of origin for at least a subset of primary skeletal tumors. Here, we analyze osteosarcomas via immunohistochemistry for known markers of CAR cells such as leptin receptor (LEPR), B‐cell factor 3 (EBF3), CXCL12, and platelet‐derived growth factor receptor alpha (PDGFRA). A large proportion of high‐grade tumors expressed LEPR, PDGFRA, and EBF3 but not CXCL12. These data raise the hypothesis that CAR cells are the cell of origin of this osteoblastic osteosarcoma subset, a finding with implications for the cellular oncogenesis of primary osteosarcoma and the development of effective targeted therapies. © 2021 The Authors. *JBMR Plus* published by Wiley Periodicals LLC on behalf of American Society for Bone and Mineral Research.

## 1 Introduction

Osteosarcoma accounts for approximately 1% of all cancer cases in the United States, making it the most common primary malignant bone tumor.^(^ ^1^, ^2^ ^)^ Survival for patients with recurrent and metastatic osteosarcoma has not changed over the past 30 years with a low 5‐year survival rate of approximately 20%.^(^ ^3^, ^4^ ^)^ Although precision medicine–targeted agents and immunotherapies have aided in the treatment of other cancers, the standard of care for osteosarcoma is still limited to combination chemotherapy and surgical resection, approaches that date back to the 1980s.^(^ ^4^, ^5^ ^)^ Identifying the lineage of cells responsible for osteosarcomas will be a critical step toward elucidating the mechanism of oncogenesis and developing effective, targeted therapies that are based on this underlying biology.

The human skeleton serves to protect vital organs throughout the body while serving an equally important role for hematopoiesis and mineral storage. Skeletal stem cells are responsible for differentiating into osteoblasts, adipocytes, and chondrocytes and are largely sufficient to recruit and organize the other cell types participating in the bone microenvironment.^(^ ^6^, ^7^ ^)^ Recent work identifies that skeletal stem cells and progenitors are not a monolithic group but rather include several distinct cell types, each of which have different signature locations, functions, and transcriptional profiles.^(^ ^8^, ^9^, ^10^ ^)^ These discrete sets of skeletal progenitors include CXC chemokine ligand (CXCL)12‐abundant reticular (CAR) cells. CAR cells are specialized, long‐lived skeletal cells that regulate the bone marrow hematopoietic niche through the expression of cytokines and growth factors. CAR cells are defined by their expression of leptin receptor (LEPR), Cxcl12, and early B‐cell factor 3 (EBF3).^(^ ^11^, ^12^ ^)^ PDGFRA is expressed by CAR cells but also other populations as well.^(^ ^13^ ^)^ In addition to orchestrating the bone marrow niche, these cells have the capacity to move from a largely quiescent basal state to differentiate into osteoblasts, especially in response to skeletal injury.^(^ ^9^, ^10^ ^)^ The signature cytokine of CAR cells is CXCL12, which binds CXCR4 and activates signaling cascades involved in cell proliferation, migration, and secretion.^(^ ^14^ ^)^ This CXCL12/CXCR4 axis directs leukocyte trafficking and hematopoietic homeostasis. Osteosarcomas downregulate CXCL12, compromising leukocyte trafficking and allowing for tumor growth and metastasis.^(^ ^15^ ^)^ Accordingly, strong expression of CXCL12 in osteosarcoma is associated with favorable prognosis.^(^ ^16^ ^)^

In this study, we sought to generate insight into whether cellular subsets of primary skeletal osteosarcoma express markers of skeletal stem or progenitor populations by staining a panel of tumors via immunohistochemistry for the defining markers of selected skeletal stem and progenitor lineages.

## 2 Materials and Methods

### 2.1 Case identification and selection

This study was approved by the Institutional Review Board of Memorial Sloan‐Kettering (MSKCC) and done in accordance with the U.S. Common Rule. A total of 16 osteosarcoma samples were taken from patients treated at MSKCC between 2000 and 2020. Diagnosis of osteosarcoma was confirmed by sarcoma pathologists, with the total set of specimens including nine high‐grade osteosarcomas and seven low‐grade parosteal osteosarcoma. Non‐cancerous bone from a clavicle fracture was utilized as a control for all stains and analyses.

### 2.2 Immunohistochemical staining

Human osteosarcoma samples were fixed in 10% neutral‐buffered formalin. Samples were processed via routine decalcification. Sectioning and staining (hematoxylin and eosin, LEPR, EBF3, CXCL12, PDGFRA) were performed by the Center for Translational Pathology at Weill Cornell using a previously published methodology.^(^ ^17^ ^)^ Commercially available antibodies were used for immunohistochemical (IHC) analysis: LEPR (ab104403, Abcam, Cambridge, MA, USA), PDGFRA (ab203491, Abcam), EBF3 (ab207705, Abcam), CXCL12 (sc‐74271, Santa Cruz, Dallas, TX, USA). Slides were analyzed using light microscopy and were graded using a negative (−), positive (+/++), strongly positive (+++) system.

### 2.3 Statistical analysis

Mann–Whitney test was conducted to compare high‐grade and low‐grade osteosarcomas following semiquantitative scoring for IHC signal intensity. Statistical analysis was performed using GraphPad Prism 8 (GraphPad Software, La Jolla, CA, USA).

## 3 Results

**Fig. 1:**
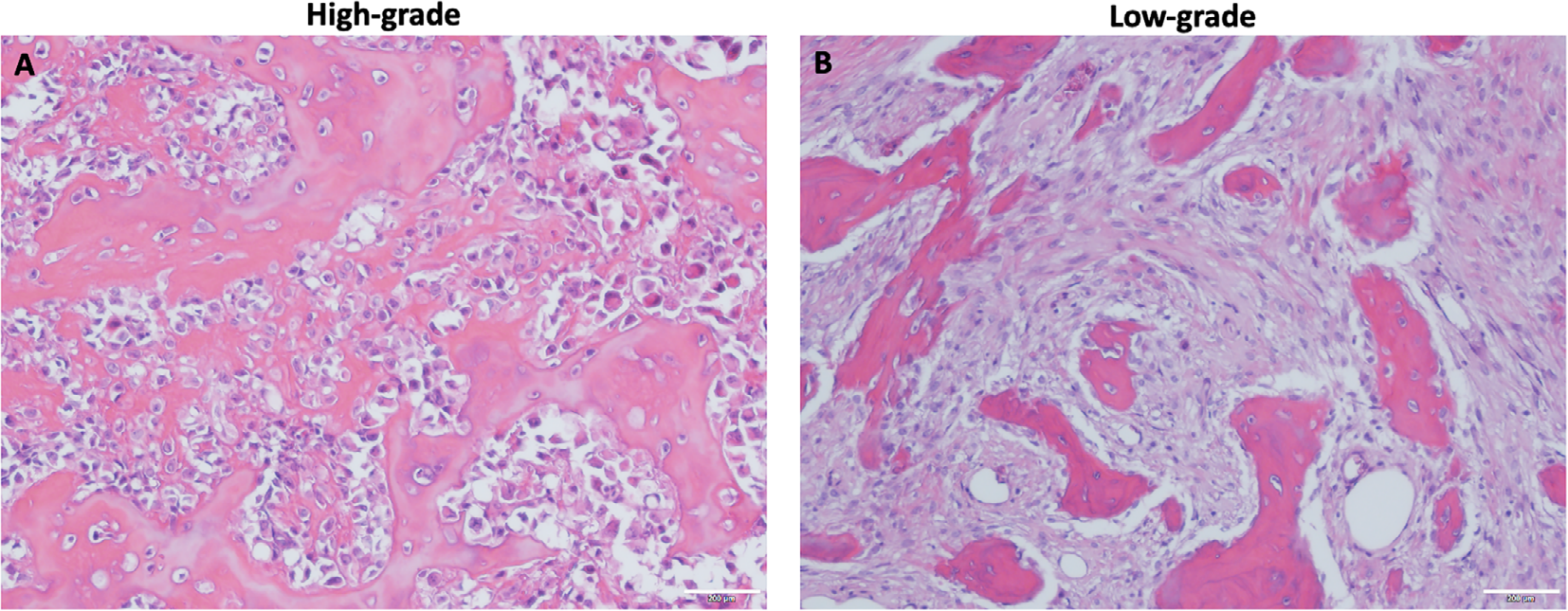
Representative images of primary osteosarcoma (OS). Hematoxylin and eosin from (*A*) high‐grade OS and (*B*) low‐grade OS. Images are representative of 8 high‐grade and 7 low‐grade tumors.

Fifteen cases of primary osteosarcoma were analyzed for established markers associated with CAR cells (LEPR, EBF3, CXCL12, PDGFRA) and subdivided into low‐grade parosteal (7/16) or high‐grade intramedullary (9/16) tumors (Fig. 1, Table 1).

**Table 1:** Osteosarcoma Expression Summary for LEPR, PDGFRA, CXCL12, and EBF3

		Chemo	Site	LEPR	PDGFRA	CXCL12	EBF3
OS‐MG 1	High‐grade	Yes	13	–	–	QNS	–
OS‐MG 2		No	Humerus	++	+++	QNS	+
OS‐MG 3		No	Femur	+	++	QNS	+
OS‐MG 6		Yes	Humerus	+	–	–	+
OS‐MG 7		Yes	Femur	+	+	–	+
OS‐MG 10		No	Femur	+	–	–	–
OS‐MG 17		Yes	Femur	++	–	–	+
OS‐MG 16		Yes	Clavicle	++	++	–	++
OS‐MG 18		Yes	Tibia	+	++	–	–
OS‐MG 11	Low‐grade	No	Femur	–	–	–	–
OS‐MG 12		No	Femur	+	–	–	++
OS‐MG 13		No	Femur	+	–	++	+++
OS‐MG 14		No	Humerus	–	+	+	–
OS‐MG 15		No	Femur	–	–	–	–
OS‐MG 4		No	Humerus	–	+	QNS	–
OS‐MG 5		No	Femur	–	–	QNS	–

QNS = quantity not sufficient to conduct the indicated stain.

Displayed are the results of semiquantitative scoring (–, +, ++, or +++, depending on signal intensity) for staining of the indicated protein markers across the indicated cases (LEPR *p* = 0.01; PDGFRA *p* = 0.15; CXCL12 *p* = 0.18; EBF3 *p* = 0.22).

**Fig. 2:**
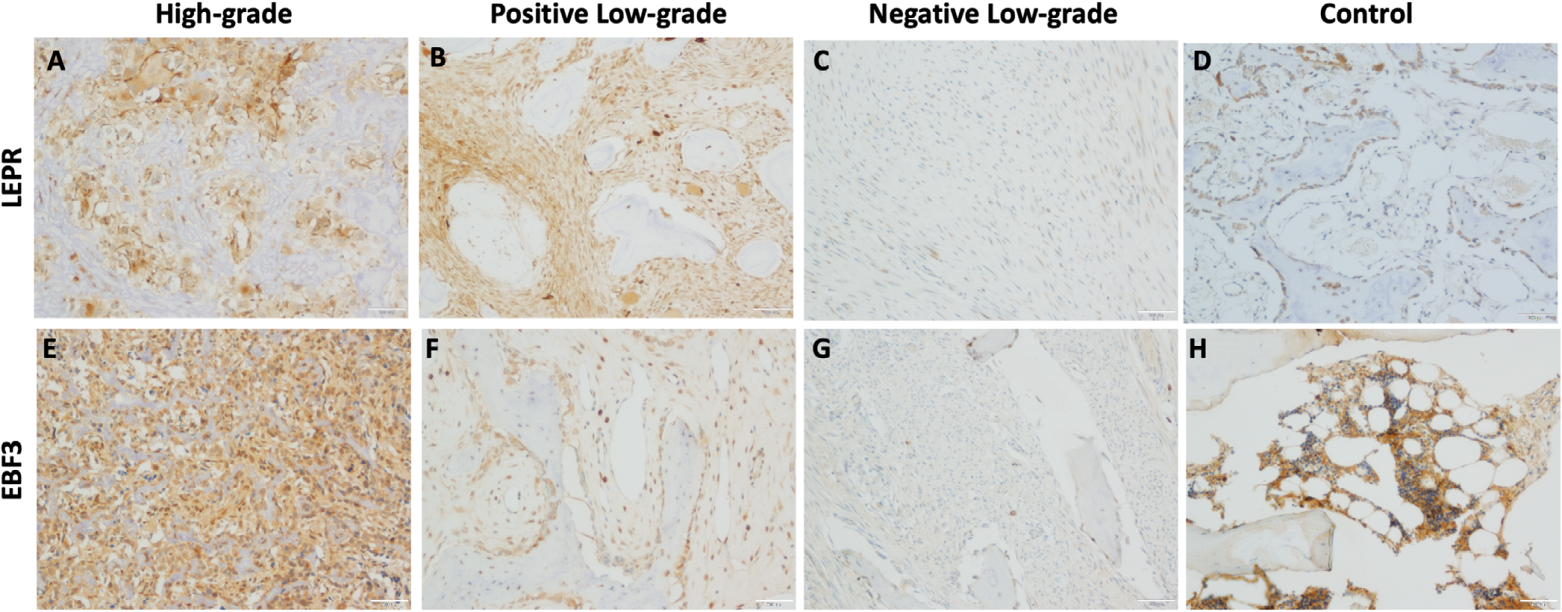
A subset of osteosarcoma (OS) expresses LEPR and EBF3. Immunohistochemical (IHC) staining was performed for LEPR and EBF3. Displayed are representative images of LEPR IHC in (*A*) high‐grade OS, (*B*) positive low‐grade OS, (*C*) negative low‐grade, and (*D*) a clavicle fragment control. Representative images of EBF3 IHC in (*E*) high‐grade OS, (*F*) positive low‐grade OS, (*G*) negative low‐grade OS, and (*H*) a clavicle fragment control.

Immunohistochemistry for LEPR revealed a high propensity (8/9) for high‐grade osteosarcomas to be LEPR+, whereas low‐grade tumors displayed a lower rate of LEPR positivity (2/7) (*p* = 0.01; Fig. 2*A*–*C*) with signal predominantly and most intensely in tumor fibroblasts. EBF3 and LEPR followed the same expression pattern across low‐ and high‐grade tumors with nearly all LEPR+ osteosarcomas (6/8) also expressing EBF3. Overall, 6/9 high‐grade and 2/7 low‐grade tumors were EBF3+ (*p* = 0.22; Fig. 2 *D*, *E*). Expression of this set of CAR cell–associated markers appeared to largely co‐occur, as LEPR, PDGFRA, and EBF3 showed a high degree of co‐occurrence in every LEPR‐positive case except one displaying only co‐occurrence of EBF3.

**Fig. 3:**
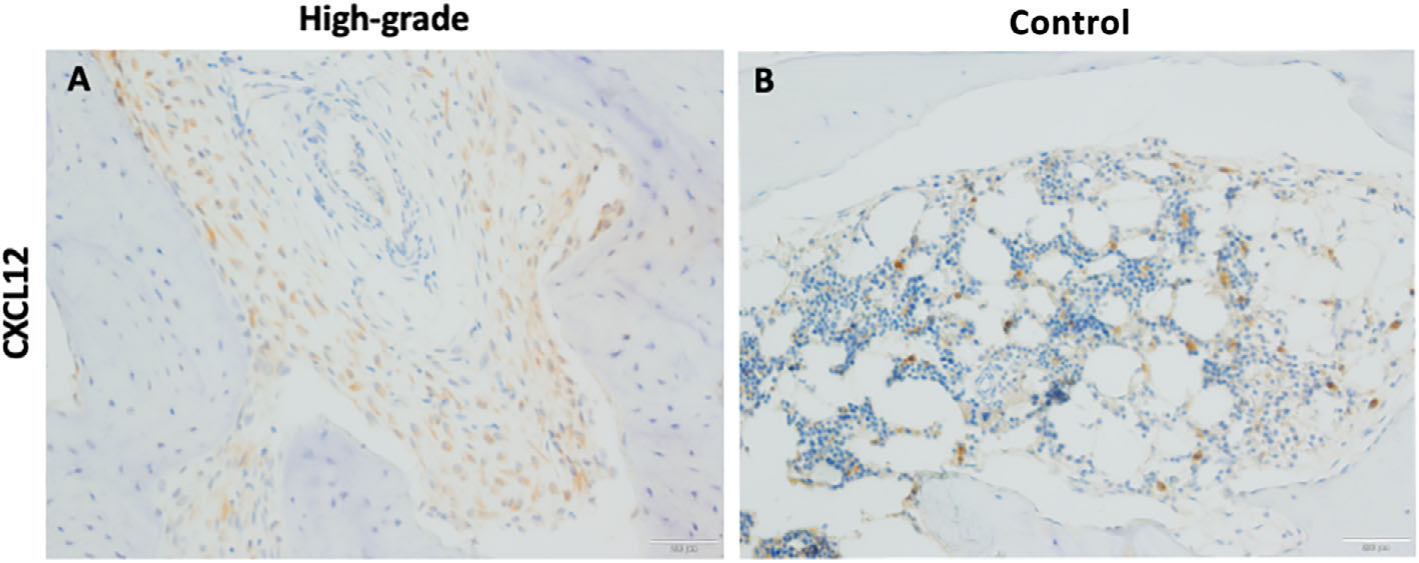
Modest CXCL12 immunohistochemical staining in OS. Two of five (*A*) low‐grade OS specimens displayed CXCL12 staining. Also displayed is a representative image of positive staining of native stromal CXCL12‐positive CAR cells in a (*B*) control clavicle fragment sample. Zero of six high‐grade OS specimens displayed CXCL12 staining.

**Fig. 4:**
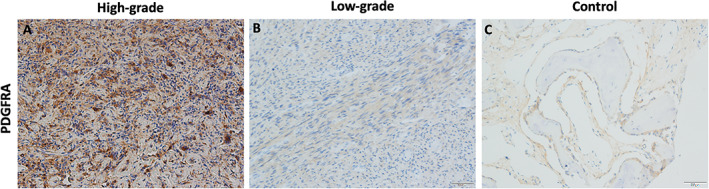
A subset of predominantly high‐grade osteosarcoma expresses PDGFRA. PDGFRA immunohistochemistry was performed on (*A*) high‐grade osteosarcoma (OS) (5/9 positive), (*B*) low‐grade (2/7 positive) OS, or (*C*) a clavicle fragment. Displayed are representative images of positive staining.

Interestingly, CXCL12 expression was not detected via IHC in any high‐grade osteosarcomas but was appreciated in 2/5 low‐grade tumors (*p* = 0.18; Fig. 3*A*). There was not sufficient tissue quantity to assess CXCL12 expression in three high‐grade and two low‐grade osteosarcoma tumors. CXCL12 staining of clavicle fractures showed a homogenous distribution of CXCL12 confined to the bone marrow stroma (Fig. 3*B*). PDGFRA IHC, on the other hand, revealed robust staining in 6/9 high‐grade and 2/7 low‐grade tumors with 4/6 tumors expressing both PDGFRA and LEPR (*p* = 0.15; Fig. 4 *A*, *B*). PDGFRA clavicle fracture staining highlighted diffusely positive signal from stromal cells adjacent to bone matrix (Fig. 4*C*). Every PDGFRA‐positive specimen except one low‐grade example also concurrently expressed LEPR.

## 4 Discussion

A critical step in improving the understanding of osteosarcoma lies in identifying the cell of origin for these tumors. Here, the hematopoietic system serves as a well‐established template, where expression of well‐established lineage‐defining markers provides a cell of origin–based classification system for leukemias and lymphomas. CXCL12‐abundant reticular (CAR) cells have been characterized as a quiescent population of perivascular stromal cells that first emerge sometime during adolescence and contribute to adult bone formation. Either in response to injury or aging, these cells differentiate into osteoblasts or adipocytes.^(^ ^9^, ^12^ ^)^ CAR cells have also been identified in human long bones and were found to have high expression of CXCL12, LEPR, and EBF3.

In this study, we show the abundant expression of the CAR cell–associated surface marker LEPR, and the characteristic transcription factor associated with CAR cells, EBF3, in a subset of human osteosarcoma samples. The expression of these markers raises the possibility of a CAR cell lineage origin for these tumors;^(^ ^12^ ^)^ however, important limitations must be acknowledged. Observational immunohistochemistry is unable to assign lineage status, and many tumors display an aberrant gain of expression in the markers not present in the physiologic cell of origin. Thus, it is also possible that the marker expression observed here instead reflects aberrant induction of a CAR cell‐like gene expression program in a non‐CAR cell lineage skeletal cell. This latter possibility would also be potentially important as convergent induction of such a gene expression program across tumors may indicate a selective advantage conferred by this gene expression program.

Genetic studies using inducible and conditional knockout alleles in mice provide evidence both that EBF3 is a defining marker for CAR cells and that EBF3 is required for CAR cell function.^(^ ^12^, ^18^ ^)^ LEPR has not yet been described as a marker of osteosarcoma despite a growing body of literature showing the importance of these cells in human and murine bone.^(^ ^19^, ^20^ ^)^ Although this study solely focuses on immunohistochemistry of these tumors, the persistence of these lineage‐defining markers in a subset of osteosarcoma suggests that this subset of osteosarcoma may be derived from transformation of CAR cells. This suggests that sorting premalignant CAR cells, especially in patients with an increased risk of osteosarcoma, will be important to identify molecular, cytologic, or histologic CAR cell precursor lesions and therefore provide further evidence regarding the cell of origin for these tumors.

Notably, CXCL12 expression did not here track together with the expression of the other CAR cell–associated markers LEPR and EBF3. Likewise, while a subset of osteosarcoma here expresses CAR cell–associated markers, they have lost many other features of CAR cells, such as their typical reticular morphology and pattern of distribution throughout the marrow. These alterations in cellular morphology and distribution may reflect transformation induced changes and emphasize that while these tumors express some CAR cell–associated markers, they lack many CAR cell defining features.

The etiology for CXCL12 expression not tracking with the expression of other CAR cell–associated markers is unclear, but literature in other tumor types suggests that loss of CXCL12 may confer a selective advantage. Aoki and colleagues found that CAR cells from patients with high leukemic burden expressed reduced levels of CXCL12 relative to patients with low leukemic burden.^(^ ^11^ ^)^ It has also been suggested that the CXCL12/CXCR4 axis serves a key role in the progression and metastasis of osteosarcomas and other cancers.^(^ ^21^, ^22^, ^23^ ^)^ Primary osteosarcoma tumors have also been shown to downregulate CXCL12 expression through an epigenetic mechanism to escape the bone and metastasize to other tissues.^(^ ^15^ ^)^ This emphasizes that evaluation of a broader signature of CAR cell–associated markers beyond staining for CXCL12 itself is necessary for the immunophenotyping of human primary skeletal tumors. The findings here also raise the issue that future studies on the prognostic significance of CXCL12 expression in osteosarcoma may need to deconvolute the possibility that CXCL12 may be associated with a distinct subset of disease derived from CAR cells from the impact of varying levels of CXCL12 expression. It will be important in future extensions of this work to evaluate whether these markers are retained in metastases.

The anatomic localization of these discrete skeletal stem and progenitor cell populations is critical to their function and can serve as a framework for analyzing osteosarcoma subsets. Our data show that low‐grade parosteal tumors were mostly CXCL12 negative, supporting the understanding that CAR cells reside in the perivascular niche of central marrow compartment. Although a small subset of LEPR cells have been reported to be periosteal stem/progenitor cells in the outer layer of the periosteum, the general lack of LEPR expression in low‐grade parosteal tumors suggests that there may be another periosteal progenitor population driving oncogenesis.^(^ ^24^ ^)^ However, as an important limitation of the specimen cohort studied here, low tumor grade and periosteal anatomic location co‐occurred, making it not possible to disentangle the effects of these parameters on the rate of positivity for CAR cell–associated markers.

Improvement in prognosis and development of new treatments for osteosarcoma have plateaued over the past 30 years. A previous study showed that conditional deletion of PDGFRA in LEPR+ stromal cell cells and treatment with imatinib in a murine model of primary myelofibrosis suppressed LEPR+ cell expansion and ameliorated bone marrow fibrosis.^(^ ^25^ ^)^ Our data add to literature showing that PDGFRA is coexpressed on LEPR cells and suggests that targeting PDGFRA may be of particular interest in this subset of disease.^(^ ^10^, ^26^ ^)^ Additionally, a previous study identified a PDGFRA‐amplified subset of osteosarcoma, raising the possibility that markers such as LEPR or EBF3 will have utility in identifying the PDGFRA‐amplified subset of osteosarcoma for therapeutic targeting.^(^ ^25^ ^)^ Pharmacologic targeting of the LEPR pathway is also a potentially attractive therapeutic strategy for this subset of osteosarcoma.^(^ ^27^ ^)^

Perhaps most importantly, these findings suggest that a broader cell of origin–based nosology for primary skeletal tumors is possible and that other skeletal progenitor populations, including cathepsin K (CTSK)‐lineage periosteal stem cells and parathyroid hormone‐related protein (PTHrP)‐positive growth plate resident stem cells, may each have characteristic associated neoplasms.^(^ ^8^, ^9^ ^)^ Both further elucidation of the markers of these subsets of skeletal stem cells and application of these markers to human primary skeletal tumor specimens will be needed to extend the results here to identify neoplastic counterparts for other skeletal cell types. In addition to enabling further clinical study of this LEPR+ subset of osteosarcoma, it should be noted that each of these cell types, including LEPR+ cells, have one or more cre lines that allow for conditionally targeting these cells in a murine preclinical model. Knowledge of the specific cell of origin for these tumors would enable the development of novel preclinical models targeted to each of these specific lineages, thereby allowing for investigation of the differences in oncogenesis and therapeutic response as driven by the biology of these distinct lineages.

## Disclosures

Authors have no conflicts of interest

5

### PEER REVIEW

The peer review history for this article is available at https://publons.com/publon/10.1002/jbm4.10596.

## References

[1] Fletcher CDM , Hogendoorn P , Mertens F . WHO classification of tumours of soft tissue and bone. 4th ed. Philadelphia, PA: Elsevier; 2013 pp 281‐295.

[2] Eilber FR . Adjuvant chemotherapy for osteosarcoma. Semin Oncol. 1989;16(4):312‐322. 2667146

[3] Meyers PA , Healey JH , Chou AJ , et al. Addition of pamidronate to chemotherapy for the treatment of osteosarcoma. Cancer. 2011;117(8):1736‐1744. 21472721 10.1002/cncr.25744 PMC3059356

[4] Link MP , Goorin AM , Miser AW , et al. The effect of adjuvant chemotherapy on relapse‐free survival in patients with osteosarcoma of the extremity. N Engl J Med. 1986;314(25):1600‐1606. 3520317 10.1056/NEJM198606193142502

[5] Bernthal NM , Federman N , Eilber FR , et al. Long‐term results (>25 years) of a randomized, prospective clinical trial evaluating chemotherapy in patients with high‐grade, operable osteosarcoma. Cancer. 2012;118(23):5888‐5893. 22648705 10.1002/cncr.27651

[6] Chan CK , Seo EY , Chen JY , et al. Identification and specification of the mouse skeletal stem cell. Cell. 2015;160(1–2):285‐298. 25594184 10.1016/j.cell.2014.12.002 PMC4297645

[7] Chan CKF , Gulati GS , Sinha R , et al. Identification of the human skeletal stem cell. Cell. 2018;175(1):43‐56.e21. 30241615 10.1016/j.cell.2018.07.029 PMC6400492

[8] Debnath S , Yallowitz AR , McCormick J , et al. Discovery of a periosteal stem cell mediating intramembranous bone formation. Nature. 2018;562(7725):133‐139. 30250253 10.1038/s41586-018-0554-8 PMC6193396

[9] Matsushita Y , Nagata M , Kozloff KM , et al. A Wnt‐mediated transformation of the bone marrow stromal cell identity orchestrates skeletal regeneration. Nat Commun. 2020;11(1):332. 31949165 10.1038/s41467-019-14029-w PMC6965122

[10] Zhou BO , Yue R , Murphy MM , et al. Leptin‐receptor‐expressing mesenchymal stromal cells represent the main source of bone formed by adult bone marrow. Cell Stem Cell. 2014;15(2):154‐168. 24953181 10.1016/j.stem.2014.06.008 PMC4127103

[11] Aoki K , Kurashige M , Ichii M , et al. Identification of CXCL12‐abundant reticular cells in human adult bone marrow. Br J Haematol. 2021;193(3):659‐668. 33837967 10.1111/bjh.17396 PMC8252541

[12] Seike M , Omatsu Y , Watanabe H , Kondoh G , Nagasawa T . Stem cell niche‐specific Ebf3 maintains the bone marrow cavity. Genes Dev. 2018;32(5–6):359‐372. 29563184 10.1101/gad.311068.117 PMC5900710

[13] Pinho S , Lacombe J , Hanoun M , et al. PDGFRα and CD51 mark human nestin+ sphere‐forming mesenchymal stem cells capable of hematopoietic progenitor cell expansion. J Exp Med. 2013;210(7):1351‐1367. 23776077 10.1084/jem.20122252 PMC3698522

[14] Neklyudova O , Arlt MJ , Brennecke P , et al. Altered CXCL12 expression reveals a dual role of CXCR4 in osteosarcoma primary tumor growth and metastasis. J Cancer Res Clin Oncol. 2016;142(8):1739‐1750. 27300512 10.1007/s00432-016-2185-5 PMC11819428

[15] Li B , Wang Z , Wu H , et al. Epigenetic regulation of CXCL12 plays a critical role in mediating tumor progression and the immune response in osteosarcoma. Cancer Res. 2018;78(14):3938‐3953. 29735547 10.1158/0008-5472.CAN-17-3801

[16] Baumhoer D , Smida J , Zillmer S , et al. Strong expression of CXCL12 is associated with a favorable outcome in osteosarcoma. Mod Pathol. 2012;25(4):522‐528. 22173290 10.1038/modpathol.2011.193

[17] Greenblatt MB , Park KH , Oh H , et al. CHMP5 controls bone turnover rates by dampening NF‐κB activity in osteoclasts. J Exp Med. 2015;212(8):1283‐1301. 26195726 10.1084/jem.20150407 PMC4516796

[18] Rodger EJ , Chatterjee A , Stockwell PA , Eccles MR . Characterisation of DNA methylation changes in EBF3 and TBC1D16 associated with tumour progression and metastasis in multiple cancer types. Clin Epigenetics. 2019;11(1):114. 31383000 10.1186/s13148-019-0710-5 PMC6683458

[19] Comazzetto S , Shen B , Morrison SJ . Restricted hematopoietic progenitors and erythropoiesis require SCF from leptin receptor + niche cells in the bone marrow. Cell Stem Cell. 2019;24(3):477‐86.e6. 30661958 10.1016/j.stem.2018.11.022 PMC6813769

[20] Sena IFG , Borges IT , Lousado L , et al. LepR+ cells dispute hegemony with Gli1+ cells in bone marrow fibrosis. Cell Cycle. 2017;16(21):2018‐2022. 28976809 10.1080/15384101.2017.1367072 PMC5731410

[21] Teicher BA , Fricker SP . CXCL12 (SDF‐1)/CXCR4 pathway in cancer. Clin Cancer Res. 2010;16(11):2927‐2931. 20484021 10.1158/1078-0432.CCR-09-2329

[22] Nervi B , Ramirez P , Rettig MP , et al. Chemosensitization of acute myeloid leukemia (AML) following mobilization by the CXCR4 antagonist AMD3100. Blood. 2009;113(24):6206‐6214. 19050309 10.1182/blood-2008-06-162123 PMC2699239

[23] Juarez J , Dela Pena A , Baraz R , et al. CXCR4 antagonists mobilize childhood acute lymphoblastic leukemia cells into the peripheral blood and inhibit engraftment. Leukemia. 2007;21(6):1249‐1257. 17410186 10.1038/sj.leu.2404684

[24] Gao B , Deng R , Chai Y , et al. Macrophage‐lineage TRAP+ cells recruit periosteum‐derived cells for periosteal osteogenesis and regeneration. J Clin Invest. 2019;129(6):2578‐2594. 30946695 10.1172/JCI98857 PMC6538344

[25] Decker M , Martinez‐Morentin L , Wang G , et al. Leptin‐receptor‐expressing bone marrow stromal cells are myofibroblasts in primary myelofibrosis. Nat Cell Biol. 2017;19(6):677‐688. 28481328 10.1038/ncb3530 PMC5801040

[26] Suehara Y , Alex D , Bowman A , et al. Clinical genomic sequencing of pediatric and adult osteosarcoma reveals distinct molecular subsets with potentially targetable alterations. Clin Cancer Res. 2019;25(21):6346‐6356. 31175097 10.1158/1078-0432.CCR-18-4032 PMC6825534

[27] Gertler A , Elinav E . Novel superactive leptin antagonists and their potential therapeutic applications. Curr Pharm Des. 2014;20(4):659‐665. 23688008 10.2174/13816128113199990014

